# Supplementary material for: Evolution of reduced minimum critical size as a response to selection for rapid pre-adult development in Drosophila melanogaster
Source: R Soc Open Sci. 2020 Jun 10;7(6):191910. doi: 10.1098/rsos.191910 (PMC7353974; doi:10.1098/rsos.191910)
Supplement: Impact of selection for accelerated pre-adult development on pre-adult mortality of Drosophila melanogaster populations, fed up to critical duration and fed up to natural pupation time [file rsos191910supp1.pdf]

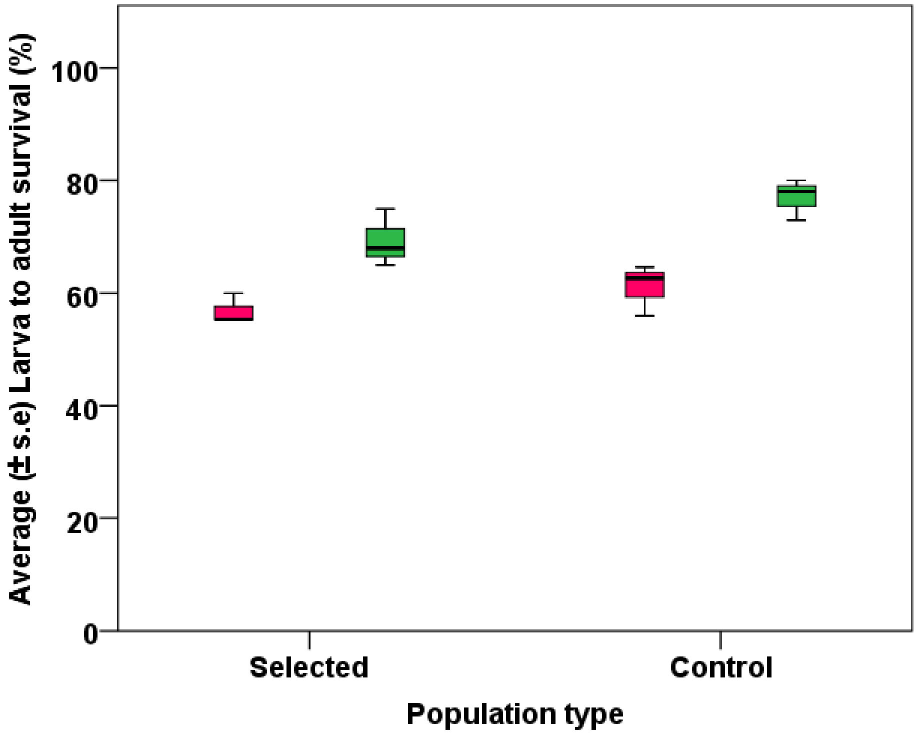


Key for colour coding

Larvae fed up to critical size time point

Larvae fed up to natural pupation time
