# Supplementary material for: Evolution of reduced minimum critical size as a response to selection for rapid pre-adult development in Drosophila melanogaster
Source: R Soc Open Sci. 2020 Jun 10;7(6):191910. doi: 10.1098/rsos.191910 (PMC7353974; doi:10.1098/rsos.191910)
Supplement: Magnitude of change in larval growth rate in the final two hours prior to attainment of critical size in selected and control populations. [file rsos191910supp2.docx]

| Population type | Average weight at critical weight time point (µg) | Average weight two hours prior to critical size time point (µg) | Growth differences | Growth rate during final 2h prior to critical size |
| --- | --- | --- | --- | --- |
| Selected population | **1002.66** | **837.7889** | **164.87** | **82.4** |
| Control population | **1308.71** | **992.3** | **316.41** | **158.2** |
